# The potential clinical and public health implications of presymptomatic genetic testing for transthyretin amyloidosis in African American/Black adults in the United States

**DOI:** 10.3389/fgene.2025.1658636

**Published:** 2025-09-15

**Authors:** Inderjeet Bharaj, Simar J. Singh, Juzer Munaim, Harneet Grewal, Sonia Sabrowsky, Andrew Boshara, Marc A. Silver, Katherine H. Brendish, Paul Underwood, Sandesh Dev, Mayowa A. Osundiji

**Affiliations:** ^1^ Abrazo Health Network Internal Medicine Residency, Glendale, AZ, United States; ^2^ University of Arizona College of Medicine- Tucson Internal Medicine Residency, Tucson, AZ, United States; ^3^ Department of Clinical Genomics, Mayo Clinic, Scottsdale, AZ, United States; ^4^ Department of Cardiology, Advanced Heart Failure and Transplant, CommonSpirit Dignity Health, St. Joseph Hospital and Medical Center, Phoenix, AZ, United States; ^5^ Phoenix and the Advanced Heart Failure/MCS/Transplant Program, Banner University Medical Center, University of Arizona, Phoenix, AZ, United States; ^6^ College of Health Solutions Arizona State University, Scottsdale, AZ, United States; ^7^ CardioMedSci, Phoenix, AZ, United States

**Keywords:** Black, transthyretin, amyloidosis, genetic, screening

## Introduction

ATTR Cardiomyopathy (ATTR-CM) is increasingly being recognized as a significant cause for heart failure in African American/Black adults (Shah et al., 2016; Muller et al., 2023; Spencer-Bonilla et al., 2021). Approximately 3.43% of African Americans harbor at least one copy of the. The *TTR* V122I allele, which is known to be associated with an increased risk of ATTR Cardiomyopathy (ATTR-CM), has a prevalence of approximately 3.43% in the African American population (Jacobson et al., 2015). It has been estimated that Black Americans will lose nearly 1 million life years due to ATTR (Selvaraj et al., 2024). Genetic testing is currently recommended to patients with a suspected clinical diagnosis of ATTR-CM (Witteles et al., 2019). With the increasing development and potential availability of drugs targeting transthyretin (Maurer et al., 2018; Rapezzi et al., 2021), debates on the possibilities of targeted presymptomatic genetic screening of vulnerable populations [including African American adults] for ATTR is emerging (Obici et al., 2016; Soper et al., 2021) and topical considering the growing need to promote health equity (Campinha-Bacote, 2024).

In this article, we report the arguments raised for and against genetic screening for ATTR in asymptomatic Black adults at a debate that was held at Arizona State University on 20 November 2023. The debate was attended by a board-certified Cardiologist, Genetic Counselors, and a Medical Geneticist, who are routinely involved in the clinical care of individuals with ATTR.

## The debate: pros and cons

Points raised in support include: the increased risk of ATTR-CM in Black Americans, the high frequency of the pathogenic *TTR* variants [especially Val122Ile (or pV142I)], and the potential benefits of presymptomatic diagnosis in facilitating preventative and therapeutic options. The challenges highlighted include: the incomplete and age-dependent penetrance of *TTR* variants, uncertainties regarding acceptability of genetic screening, the risk of genetic discrimination, the uncertain impact for presymptomatic diagnosis, the costs of screening and limitations associated with variants of uncertain significance (VUS).

Pros: Team A argued that presymptomatic screening of African Americans, can pave the way for potentially preventative approaches, awareness of symptoms, and improved surveillance for ATTR (Maurer et al., 2019). Additionally, presymptomatic screening is likely to lead to timely initiation of treatment (Ueda et al., 2020). With the United States Food and Drug Administration’s (FDA) approval of Tafamidis (Maurer et al., 2018; Hussain et al., 2025) and the growing possibilities (Costabel et al., 2025) for other preventative as well as therapeutic approaches that may alter the disease course [such as RNA-targeted therapies [like Patisiran, Eplontersen, and Inotersen (Aimo et al., 2022)] CRISPR-Cas9 gene editing [e.g., nexiguran, and ziclumeran (Fontana et al., 2024)], team A argued that presymptomatic screening will result in early interventions including therapeutic and supportive management. Moreover, cascade testing of asymptomatic family members will be a potential secondary benefit of presymptomatic screening (Ueda et al., 2020).

Team A maintained that direct-to-consumer genetic tests are already reporting the common *TTR* variants (Soper et al., 2021). Team A cited an example of a 45-year-old African American male who self-referred to genetics clinic in Arizona after a nonclinical, direct-to-consumer, genetic test revealed that he had a heterozygous *TTR* p. V122I variant, in the absence of personal or family history of symptoms related to ATTR. This case highlighted the potential for presymptomatic diagnosis in more African American patients, if opportunities for screening for common *TTR* variants are created.

Cons: Team B argued that the penetrance of ATTR appears to be incomplete as well as being subject to age-dependent penetrance (Maurer et al., 2016). In a study, involving 3,856 subjects from the Atherosclerosis Risk in Communities (ARIC) cohort, Selvaraj and colleagues reported significant increases in heart failure and mortality risk became apparent from around age 65 years in individuals harboring *TTR* p. V142I (Selvaraj et al., 2023). Approximately 150 variants in *TTR* have been identified in individuals with ATTR amyloidosis (Monda et al., 2024). Team B maintained that a comprehensive screening program for Black adults may be difficult to achieve based on the current clinical scientific knowledge of *TTR* variants due to challenges associated with variants of uncertain significance [VUS] (Mighton et al., 2023; Burke et al., 2022). The psychological, ethical, and economic consequences of VUS cannot be overemphasized (Makhnoon et al., 2019a; Makhnoon et al., 2019b; Jeanne and Chung, 2025; Favalli et al., 2021; Federici and Soddu, 2020). Team B explained that presymptomatic screening for ATTR may need to focus on established pathogenic and likely pathogenic *TTR* variants, potentially limiting the capacity of such screening program for individuals with non-founder mutations.

Team B contended that the uptake of ethnicity centered presymptomatic genetic screening might be low in Black communities for historic reasons (Brandon et al., 2005) without appropriate pre-test counseling and a well-defined post-test counseling and clinical management pathway (Skirton et al., 2013). Team B cited that the current limitations of Genetic Information Nondiscrimination Act (GINA) may also complicate presymptomatic pre-test counselling for an already vulnerable population (Prince et al., 2022). The potential costs associated with widespread presymptomatic genetic screening for Black adults were also underscored by team B. The cost effectiveness of genomic population health screening programs is a subject of ongoing investigations that is topical considering the resource constrains faced by many healthcare systems. A recent review of eight modeling studies of cost effectiveness of genomic population screening in adults for three countries [United States, United Kingdom and Australia] using quality-adjusted life year (QALYs) and incremental cost-effectiveness ratio (ICER) as well as willingness to pay (WTP) threshold suggests that genomic population health screening is likely to be cost-effective (Wildin, 2024).

## Discussion and conclusion

This debate highlights the complexities that are potentially associated with implementing routine genetic screening for ATTR in the Black population despite the growing need for the public health screening program. The panel recognized that the increased risk of ATTR cardiac amyloidosis in African American adults (Muller et al., 2023; Jacobson et al., 2015; Maurer et al., 2016) together with the challenges of racial disparities in access to care results in increased vulnerability (Muller et al., 2023). Screening approaches will need to be guided by allele frequency, penetrance, predictive value, and preventative as well as therapeutic implications.

The development of programs that can create an improved public awareness of the increased vulnerability of African Americans to ATTR-CM is increasingly becoming necessary. The results of this debate and new evidence suggest that early detection of ATTR-CM via widespread ATTR variant genetic testing in asymptomatic Black adults may potentially improve patient outcomes (Witteles et al., 2019; Maurer et al., 2018; Rapezzi et al., 2021; Obici et al., 2016), however to facilitate increased uptake of genetic testing, we believe that additional evidence generation on the risks and benefits of genetic screening of at-risk communities is required (Wildin, 2024). A recent study estimated that Black Americans will lose nearly 1 million life years due to ATTR (Selvaraj et al., 2024). The ongoing and future clinical trials may shed further light on novel approaches to delay the development of ATTR CM and/or polyneuropathy in asymptomatic patients (Garcia-Pavia et al., 2024).

The panel concluded that clinical genetic tests for the common *TTR* variants should be increasingly offered to Black adults, following appropriate risk and culturally tailored pretest counseling, considering the preventative and therapeutic benefits. The panel posits that approaches to increase the awareness of ATTR-CM in Black people will be beneficial. Pilot programs, and cost-effectiveness clinical trials, as well as culturally tailored pre and post-test counseling are therefore warranted.
